# Dexmedetomidine-ketamine versus Dexmedetomidine-midazolam-fentanyl for monitored anesthesia care during chemoport insertion: a Prospective Randomized Study

**DOI:** 10.1186/s12871-016-0211-4

**Published:** 2016-08-02

**Authors:** Eun Hee Chun, Myeong Jae Han, Hee Jung Baik, Hahck Soo Park, Rack Kyung Chung, Jong In Han, Hun Jung Lee, Jong Hak Kim

**Affiliations:** 1 Department of Anesthesiology and Pain Medicine, School of Medicine, Ewha Womans University, 1071 Anyangcheon-ro, Yangcheon-gu, Seoul, 07985 South Korea; 2 Department of Anesthesiology and Pain Medicine, CHA Gumi Medical Center, CHA University, Gumi, South Korea

**Keywords:** Dexmedetomidine, Fentanyl, Ketamine, Midazolam, Monitored anesthesia care

## Abstract

**Background:**

Dexmedetomidine as a sole agent showed limited use for painful procedures due to its insufficient sedative/analgesic effect, pronounced hemodynamic instability and prolonged recovery. The aim of this study was to compare the effects of dexmedetomidine-ketamine (DK) versus dexmedetomidine-midazolam-fentanyl (DMF) combination on the quality of sedation/analgesia and recovery profiles for monitored anesthesia care (MAC).

**Methods:**

Fifty six patients undergoing chemoport insertion were randomly assigned to group DK or DMF. All patients received 1 μg.kg^−1^ dexmedetomidine over 10 min followed by 0.2–1.0 μg.kg^−1^h^−1^ in order to maintain 3 or 4 of modified Observer's Assessment of Analgesia and Sedation score checked every 3 min. At the start of dexmedetomidine infusion, patients in group DK or DMF received 0.5 mg.kg^−1^ ketamine or 0.05 mg.kg^−1^ midazolam + 0.5 μg.kg^−1^ fentanyl intravenously, respectively. When required, rescue sedatives (0.5 mg.kg-1 of ketamine or 0.05 mg.kg-1 of midazolam) and analgesics (0.5 mg.kg-1 of ketamine or 0.5 μg.kg-1 of fentanyl) were given to the patients in DK or DMF group, respectively. The primary outcome of this study was the recovery parameters (time to spontaneous eye opening and the length of the recovery room stay). The secondary outcomes were parameters indicating quality of sedation/analgesia, cardiorespiratory variables, and satisfaction scores.

**Results:**

There were no significant differences in the onset time, time to spontaneous eye opening, recovery room stay, the incidences of inadequate analgesia, hypotension and bradycardia between the two groups. Despite lower infusion rate of dexmedetomidine, more patients in the DMF group had bispectral index (BIS) < 60 than in the DK group and vice versa for need of rescue sedatives. The satisfaction scores of patients, surgeon, and anesthesiologist in the DMF group were significantly better than the DK group.

**Conclusions:**

The DK and DMF groups showed comparable recovery time, onset time, cardiorespiratory variables, and analgesia. However, the DMF group showed a better sedation quality and satisfaction scores despite the lower infusion rate of dexmedetomidine, and a higher incidence of BIS < 60 than the DK group.

**Trial registration:**

Clinical Trial Registry of Korea KCT0000951, registered 12/12/2013

## Background

Dexmedetomidine, a selective α_2_-adrenergic receptor agonist, exerts sedative and analgesic effects without respiratory depression, unlike most analgesic/sedative drugs, such as opioids, benzodiazepines, and propofol [1]. Although well described and successful for sedation for nonpainful procedures, dexmedetomidine has been largely unsuccessful in providing adequate analgesia when used alone for painful procedures [2, 3]. To overcome these shortcomings, several agents can be used in combination with dexmedetomidine for monitored anesthesia care (MAC) during invasive procedures.

Ketamine, an N-methyl-D-aspartate receptor antagonist, is one of those adjuvant drugs due to its sedative, analgesic, and sympathomimetic effects [4]. The combination of ketamine with dexmedetomidine can serve not only to eliminate the slow onset of sedation, but also to prevent the bradycardia and hypotension that occur when dexmedetomidine is used as a sole agent [5]. There has been an increasing number of reports on the combination of ketamine with dexmedetomidine, particularly in pediatric patients [5–7]. However, there is lack of clinical data about the dexmedetomidine-ketamine combination for procedural sedation in adults.

Midazolam is another commonly-used intravenous sedative agent with a rapid onset and relatively rapid recovery compared to other benzodiazepines. However, its use in invasive procedures can be limited due to its respiratory depressive effect and lack of analgesic action. Kose et al. [8] showed that in transurethral procedures, the dexmedetomidine-midazolam combination provided satisfactory sedation but a lower analgesic effect and hemodynamic stability with a longer recovery time than the dexmedetomidine-ketamine combination. Angst et al [9] reported that dexmedetomidine lacked analgesic efficacy at doses of mild to severe sedation. Therefore, the combination of dexmedetomidine with midazolam requires additive analgesics such as opioids for painful invasive procedures.

However, until now, there is no study comparing the effects of dexmedetomidine-midazolam-fentanyl (DMF) with dexmedetomidine-ketamine (DK) combination on the quality of sedation/analgesia, hemodynamic parameters, and recovery profiles for MAC during painful procedures. We hypothesized that DK combination would be better in the recovery profiles compared with DMF combination. We evaluated the effects of DK versus DMF on sedation/analgesia, the cardiorespiratory variables, the recovery time and the satisfaction scores of the patients, surgeon, and anesthesiologist during MAC for chemoport insertion, a procedure requiring sedation and analgesia.

## Methods

This prospective randomized double-blind study was conducted on patients undergoing chemoport insertion. Ethical approval for this study (ECT 13-37A-55) was provided by the local Ethics Committee of Ewha Womans University, Seoul, Korea on 6 November 2013. The trial was registered with the Clinical Trial Registry of Korea under assigned number KCT0000951, and written informed consent was obtained from all patients.

Fifty-six patients (aged 18–65 years, American Society of Anesthesiologists physical status I or II) were enrolled and were randomly assigned to the DK group or the DMF group using a computer-generated randomization list. The exclusion criteria included patients with a known allergy to any medication used in the study, the chronic use of analgesics and/or sedatives, renal or hepatic dysfunction, psychiatric disorders, respiratory disorders, sleep apnea, or arrhythmia. None of the patients received premedication. After a baseline measurement of the blood pressure (BP), heart rate (HR), oxygen saturation by pulse oximeter (SpO_2_), respiratory rate (RR), and bispectral index (BIS) as monitored with the BIS VISTA® monitor (Aspect Medicine system Inc., Norwood, USA), the patients received 5 L.min^−1^ of oxygen through a facemask and 0.9 % NaCl at a rate of 8 ml.kg^−1^.h^−1^. A 1 μg.kg^−1^ intravenous (IV) bolus infusion of dexmedetomidine (Precedex®; Hospira, Lake Forest, IL) was administered over 10 min via a syringe pump (Terufusion TE-331®; Terumo, Tokyo, Japan). At the start of the dexmedetomidine bolus infusion, the patients in the DK or DMF group received 0.5 mg.kg^−1^ of ketamine (Ketomine; Daihan Pharm, Seoul, Korea) or 0.05 mg.kg^−1^ of midazolam (Bukwang Pharm, Seoul, Korea) + 0.5 μg.kg^−1^ of fentanyl (Hana Pharm, Seoul, Korea) intravenously, respectively. To assess the onset time of the sedation – which is defined as the period from the beginning of the administration of the study drugs to the moment when the modified Observer’s Assessment of Alertness/Sedation (OAA/S) score [10] (5 = responds readily to name spoken in normal tone, 4 = lethargic response to name spoken in normal tone, 3 = responds only after name is called loudly and/or repeatedly, 2 = responds only after mild prodding or shaking, 1 = does not respond to mild prodding or shaking) reaches 3, the modified OAA/S score was checked every 30 s. When the modified OAA/S score reached 3 [11], the surgical procedure was initiated with a subcutaneous infiltration of 1 % lidocaine (Huons, Jecheon, Korea) 15 ml at the skin incision site. After finishing the bolus infusion of dexmedetomidine, a continuous infusion was initiated at a rate of 0.6 μg.kg^−1^h^−1^ in both groups. The continuous infusion rate of dexmedetomidine was titrated with a 0.2 μg.kg^−1^h^−1^ increase or decrease up to 1.0 μg.kg^−1^h^−1^, in order to maintain a score of 3 or 4 in the modified OAA/S checked every 3 min. If the modified OAA/S score was 5 despite the use of the maximal infusion rate of dexmedetomidine (1.0 μg.kg^−1^h^−1^), 0.5 mg.kg^−1^ of ketamine or 0.05 mg.kg^−1^ of midazolam was given as a rescue drug to the patients in DK or DMF group, respectively. If the patients asked for additional analgesics, 0.5 mg.kg^−1^ of ketamine or 0.5 μg.kg^−1^ of fentanyl was given as a rescue drug in DK group or DMF group, respectively. The administration of 5 mg of ephedrine for hypotension (mean blood pressure < 60 mmHg or a decrease in systolic blood pressure > 20 %) and 0.5 mg of atropine for bradycardia (HR < 50) was planned. The following parameters were measured and recorded every 3 min: the BP, HR, SpO_2_, RR, BIS, modified OAA/S score, and the infusion rate of dexmedetomidine. Adverse events such as bradycardia, hypotension, pain complaint, and the administration of rescue drugs were also recorded. When the surgical procedure was finished, we stopped the continuous infusion of dexmedetomidine and recorded the total dose of dexmedetomidine administered. We recorded the time to spontaneous eye opening and the length of the recovery room stay. The time to spontaneous eye opening was defined as the time from the discontinuation of the sedative drugs to the time of spontaneous eye opening, and the recovery room stay was defined as the time from entrance in the recovery room to the moment a modified Aldrete’s score [12] ≥ 9 was achieved. The satisfaction scores of the patients, surgeon, and anesthesiologist were also investigated on a 5-point scale, with 5 = very satisfied, 4 = satisfied, 3 = slightly satisfied, 2 = dissatisfied, and 1 = very dissatisfied. The satisfaction score of the surgeon was investigated after surgery, and the satisfaction scores of the patients and the anesthesiologist were investigated when the patients left the recovery room. The adverse events in the recovery room were also recorded.

Ports were placed in the subclavian vein under fluoroscopic guidance by a single surgeon for all patients. One of the investigators (MJH) performed the procedural sedation and determined the satisfaction scores. The patients and surgeon did not know which group the patients had been allocated to.

The primary outcome of this study was the recovery parameters (time to spontaneous eye opening and the length of the recovery room stay). The secondary outcomes were parameters indicating quality of sedation/analgesia, cardiorespiratory variables, and satisfaction scores.

SPSS (ver. 18.0, Chicago, IL, USA) was used for the statistical analysis. The data were expressed as numbers, percentages, or mean ± standard deviation (SD). The categorical data were analyzed with a chi-square test or Fisher’s exact test, as appropriate. The differences in continuous parameters between the two groups were analyzed using an unpaired student *t*-test. A repeated measures analysis of variance was used to test the difference between the two groups in terms of percentage change in the blood pressure and heart rate, modified OAA/S score, infusion rate of dexmedetomidine, and BIS over time. A P value < 0.05 was considered statistically significant. Using a two-sided design at a significance level of 5 % with a power of 90 %, an estimated 23 patients per group were needed to detect a sufficient effect size based on previous data [7]. Assuming a 20 % dropout rate, we designed the study with 28 patients in each group.

## Results

**Fig. 1:**
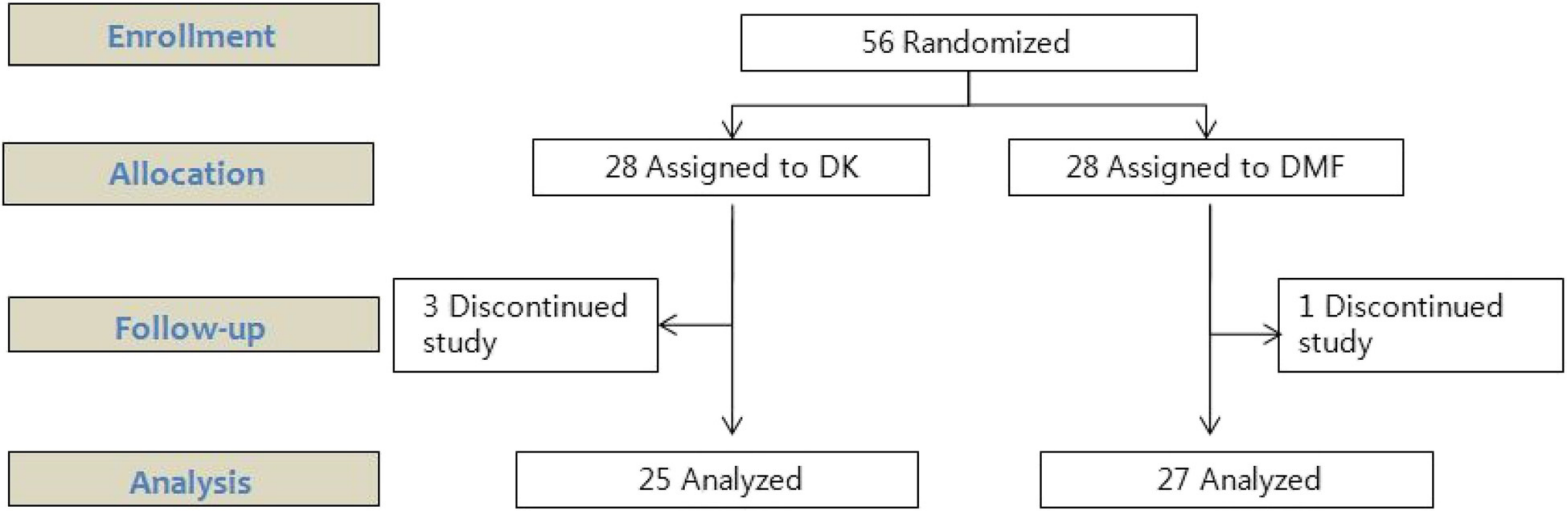
Flow chart of patient enrollment

Fifty-six patients were enrolled in the study. This study was performed between November 2013 and October 2014. Three patients in the DK group and one in the DMF group were excluded from the data analysis due to protocol violations (Fig. 1). The demographic data and initial vital signs were comparable between the two groups (Table 1).

**Table 1:** Demographic and preoperative data in patients receiving dexmedetomidine-ketamine or dexmedetomidine-midazolam-fentanyl

	Group DK (*n* = 25)	Group DMF (*n* = 27)
Age (yrs)	50.6 ± 9.8	46.7 ± 6.2
Sex (M/F)	3/22	1/26
Height (cm)	159.2 ± 6.2	159.3 ± 6.3
Weight (kg)	58.8 ± 9.1	59.3 ± 7.0
ASA physical status(I/II)	20/5	21/6
MBP (mmHg)	89 ± 14	92 ± 18
HR (beats/min)	75 ± 15	74 ± 9
SpO_2_ (%)	100 ± 0.0	100 ± 0.3
Respiratory Rate	19.6 ± 0.8	19.5 ± 0.9

Data are presented as number or mean ± SD. There are no significant differences between the two groups

*ASA* American Society of Anesthesiologists, *group DK* dexmedetomidine-ketamine group, *group DMF* dexmedetomidine-midazolam-fentanyl group, *MBP* mean blood pressure, *HR* heart rate, *SpO*_*2*_ hemoglobin oxygen saturation by pulse oximeter

The time to spontaneous eye opening (6.52 ± 10.68 vs. 6.59 ± 10.86 min) and the length of the recovery room stay (11.48 ± 11.23 vs. 12.33 ± 11.85 min for DK and DMF groups, respectively) were not significantly different between the two groups. One patient in each group required rescue drug for hypotension in recovery room. They also needed rescue drug for hypotension during MAC. None of the patients needed delayed discharge from the RR for hemodynamic and respiratory complications or psychotropic reactions.

There was no significant difference in the onset time of sedation between the two groups (DK group: 6.98 ± 6.33 vs. DMF group: 5.94 ± 4.40 min) (Table 2). Although there was no significant difference in the number of patients requiring rescue analgesics {6 (24.0 %) in the DK group and 4 (14.8 %) in the DMF group}, the number of patients requiring rescue sedatives was significantly higher in the DK group {6 (24.0 %)} than in the DMF group {0 (0 %)} (Table 2). The lowest OAA/S score for all patients in the study was 2, and the number of patients showing an OAA/S score of 2 at least once in the course of the study was 21 (77.8 %) in the DMF group, which was significantly higher than in the DK group {10 (40 %)} (Table 2). There were no significant differences between the two groups in the number of patients requiring rescue drugs for hypotension or bradycardia, the duration of monitored anesthesia care, or the total dose of dexmedetomidine (Table 2).

**Table 2:** Parameters regarding to quality of sedation/analgesia

	Group DK (*n* = 25)	Group DMF (*n* = 27)	P value
Onset time of sedation (min)	6.98 ± 6.33	5.94 ± 4.40	0.50
Patient requiring rescue sedatives [n (%)]	6 (24.0 %)	0 (0.0 %)*	0.009
Patient requiring rescue analgesics [n (%)]	6 (24.0 %)	4 (14.8 %)	0.492
Modified OAA/S score = 2 [n (%)]	10 (40.0 %)	21 (77.8 %)*	0.006
Duration of MAC (min)	28.8 ± 5.5	28.6 ± 4.4	0.903
Total dose of dexmedetomidine (μg)	102.2 ± 24.3	100.6 ± 14.2	0.770
Patient requiring rescue drug for hypotension [n (%)]	7 (28.0 %)	3 (11.1 %)	0.167
Patient requiring rescue drug for bradycardia [n (%)]	3 (12.0 %)	0 (0.0 %)	0.104

Data are presented as number (%) or mean ± SD

*P < 0.05, compared with group DK

*Group DK* dexmedetomidine-ketamine group, *group DMF* dexmedetomidine-midazolam-fentanyl group, *modified OAA/S score* modified Observer’s Assessment of Analgesia and Sedation score, *MAC* monitored anesthesia care

We analyzed the data on the change in mean blood pressure (MBP), HR, modified OAA/S score, infusion rate of dexmedetomidine, and BIS up to 21 min after the start of the sedation, as many patients had missing values after that time due to the short duration of the procedure. The interaction between time and group was significant, so we could not summarize the overall effect and we presented the differences between individual time points or between groups.

**Fig. 2:**
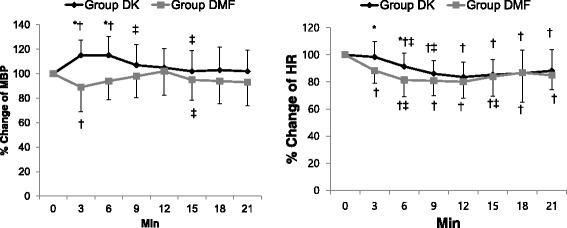
The change of hemodynamic variables, mean blood pressure (MBP) (A) and heart rate (HR) (B). Data are presented as mean ± SD. Group DK = dexmedetomidine-ketamine group; group DMF = dexmedetomidine-midazolam-fentanyl group, *: *P* < 0.05, compared with group DMF. †: *P* < 0.05, compared with baseline value. ‡: *P* < 0.05, compared with previous value

There were significant differences between the two groups in the percentage change in MBP and HR only 3 and 6 min after the onset of the sedative drug administration (Fig. 2).

**Fig. 3:**
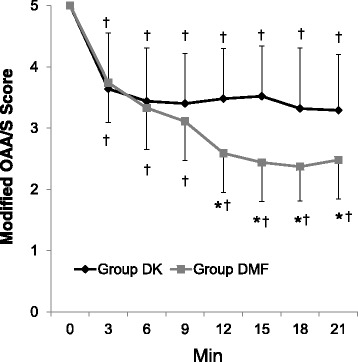
The change of Modified Observer’s Assessment of Alertness/Sedation (OAA/S) score. Data are presented as mean ± SD. Group DK = dexmedetomidine-ketamine group; group DMF = dexmedetomidine-midazolam-fentanyl group, *: *P* < 0.05, compared with group DMF. †: *P* < 0.05, compared with baseline value

**Fig. 4:**
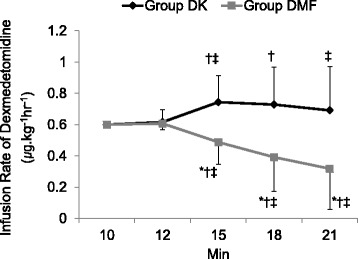
The change of infusion rate of dexmedetomidine (μg/kg/h). Data are presented as mean ± SD. Group DK = dexmedetomidine-ketamine group; group DMF = dexmedetomidine-midazolam-fentanyl group, *: *P* < 0.05, compared with group DMF. †: *P* < 0.05, compared with baseline value, ‡: *P* < 0.05, compared with previous value

**Fig. 5:**
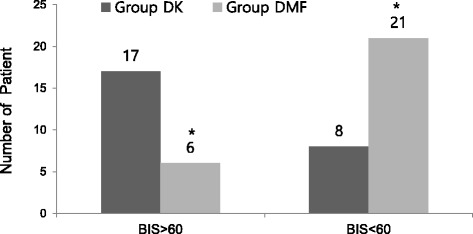
The distribution of the patients showing bispectral index (BIS) < 60 at least once during the study in both groups. Data are presented as number of patient. The number of patients showing BIS < 60 at least once in group DMF {21 (77.8 %)} was significantly more than that in group DK {8 (32 %)}. *: *P* < 0.05, compared with group DK

**Fig. 6:**
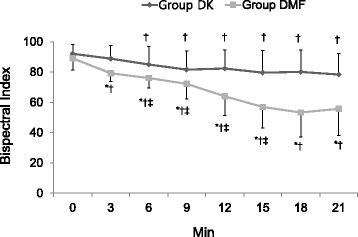
The change of bispectral index in both groups. Data are presented as mean ± SD. Group DK = dexmedetomidine-ketamine group; group DMF = dexmedetomidine-midazolam-fentanyl group, *: *P* < 0.05, compared with group DMF. †: *P* < 0.05, compared with baseline value, ‡: *P* < 0.05, compared with previous value

The modified OAA/S score was significantly lower in the DMF group than in the DK group from 12 to 21 min after the onset of the drug administration (Fig. 3). Despite the lower infusion rate of dexmedetomidine, the number of patients showing a BIS < 60 at least once in the DMF group {21 (77.8 %)} was significantly higher than in the DK group {8 (32 %)} (Figs. 4 and 5). The mean BIS values were also significantly higher in the DK group than in the DMF group (Fig. 6).

No respiratory depression (respiratory rate < 9) or hemoglobin oxygen desaturation (SpO_2_ < 93 %) was observed in the patients. None of the patients required airway opening maneuvers such as jaw thrust or chin lift during the study.

**Fig. 7:**
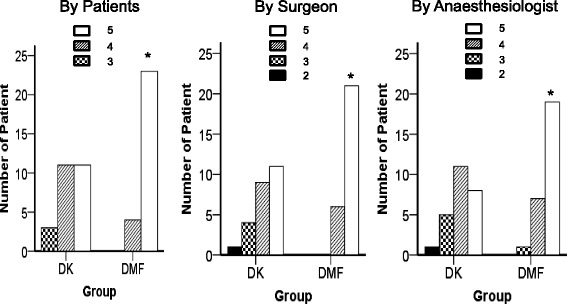
Satisfaction score by patients (A), surgeon (B), and anesthesiologist (C) (5-point scale, with 5 = very satisfied, 4 = satisfied, 3 = slightly satisfied, 2 = dissatisfied, and 1 = very dissatisfied). Data are presented as number of patient. The satisfaction scores by patients, surgeon, and anesthesiologist in group DMF were significantly better than group DK. Group DK = dexmedetomidine-ketamine group; group DMF = dexmedetomidine-midazolam-fentanyl group, *: *P* < 0.05, compared with group DK

The satisfaction scores of the patients, surgeon, and anesthesiologist were significantly better in the DMF group than in the DK group (Fig. 7). The number of patients with a satisfaction score of 5 (very satisfied) was 23 (85.2 %), 21 (77.8 %), and 19 (70.4 %) according to the patients, surgeon, and anesthesiologist, respectively, in the DMF group. The lowest satisfaction score indicated by the patients was 3, and that indicated by the surgeon and anesthesiologist was 2. Two patients in the DK group were scored as “dissatisfied”: one by the surgeon (the patient received “slightly satisfied” from the anesthesiologist and scored themselves as “satisfied”) and the other by the anesthesiologist (the patient received “slightly satisfied” from the surgeon and scored themselves as “satisfied”). However, no patient in the DMF group was scored as “dissatisfied” by the surgeon or anesthesiologist.

## Discussion

Our results demonstrate that MAC using dexmedetomidine-midazolam-fentanyl for chemoport insertion has no difference in the onset time, time to spontaneous eye opening, recovery room stay, the incidences of inadequate analgesia, hypotension and bradycardia compared with dexmedetomidine-ketamine group. The DMF group displayed better sedation quality and satisfaction scores from the patients, surgeon, and anesthesiologist despite the lower infusion rate of dexmedetomidine and higher incidence of BIS < 60 than in the DK group.

Chemoport insertion is a painful and discomforting procedure which usually requires MAC for analgesia and sedation. There have been a growing number of reports of the use of dexmedetomidine for procedural sedation. However, dexmedetomidine lacks an analgesic effect for heat and electrical pain even at doses causing severe sedation [9]. Moreover, the sole use of dexmedetomidine for colonoscopy is limited by the frequent requirement of supplemental fentanyl, profound hemodynamic instability, and prolonged recovery [2]. In particular, the co-administration of ketamine is rising to compensate for the slow onset, hypotension, bradycardia, and inadequate analgesia from the sole use of dexmedetomidine [5–8, 13]. However, meticulous care is required for using ketamine due to its adverse effects including emergence delirium, visual and auditory hallucinations, and vivid unpleasant dreams. We therefore compared the analgesic and sedative effects of the dexmedetomidine-ketamine combination with the dexmedetomidine-midazolam-fentanyl combination during MAC for chemoport insertion.

There was no difference in the mean onset time of sedation between the two groups in this study. In both groups, it was around 6–7 min, which is shorter than the minimum 10 min which is usually required when adopting loading dose of dexmedetomidine, but longer than the onset of sedation with propofol [14]. Therefore, the addition of ketamine or midazolam-fentanyl to dexmedetomidine can speed the onset of sedation from dexmedetomidine alone.

There was no difference in the number of patients requiring rescue analgesics between the two groups in this study. However, there was a significant difference in the number of patients requiring rescue sedatives. No patient required rescue sedatives in the DMF group, while six patients (24 %) in the DK group required rescue sedatives due to an OAA/S score of 5 despite a maximal infusion rate of dexmedetomidine (1.0 μg.kg^−1^h^−1^). On the other hand, more patients {21 (77.8 %)} in the DMF group showed an OAA/S score of 2 reflecting deep sedation than in the DK group {10 (40.0 %)}. To reproduce a clinical setting, we did not fix the continuous infusion rate of dexmedetomidine in this study. To achieve adequate sedation (a modified OAA/S score of 3–4), we adjusted the infusion rate up or down by 0.2 μg.kg^−1^h^−1^. We usually had to increase and decrease the continuous infusion rate of dexmedetomidine in DK and DMF groups, respectively. Therefore the infusion rate of dexmedetomidine in the DMF group was significantly lower than in the DK group from 15 to 21 min after the start of the drug administration.

The BIS in the DMF group was significantly lower than in the DK group throughout the study. The hypnotic effect of ketamine is characterized by a dissociative mechanism, ketamine-induced unconsciousness is characterized by suppression of high-frequency gamma activity and a breakdown of cortical coherence [15]. The BIS increase in response to ketamine is paradoxical in so far as the anesthesia level is deepened by the administration of an additional anesthetic agent [16]. The BIS usage has limitation to determine the anesthetic depth in MAC with ketamine, so we used the modified OAA/S for the adjustment of anesthetic depth.

However, the finding that six patients (24 %) in the DK group but none in the DMF group needed rescue sedatives implied inadequate sedation or inconvenience during the procedural sedation. This is also consistent with the finding that the satisfaction scores of the patients, surgeon, and anesthesiologist in the DMF group were significantly higher than in the DK group (Fig. 7).

The common adverse effects of dexmedetomidine are hypotension, hypertension, and bradycardia [17]. Dexmedetomidine has a biphasic effect on blood pressure, causing a decrease in the mean arterial pressure at low plasma concentrations (<1.9 ng.ml^−1^) due to vasodilation from the activation of the α_2A_ receptor, and an increase at higher plasma concentrations due to vasoconstriction from the activation of the peripheral α_2B_ receptor [18]. In a human study, a 2-min infusion of 1 or 2 μg.kg^−1^ dexmedetomidine produced an early transient increase in MBP (16 or 24 %, respectively, peak at 3 min lasting < 11 min) with a concomitantly reduced HR [19]. In our study, dexmedetomidine was administered as a 1 μg.kg^−1^ loading infusion over 10 min, followed by a continuous infusion of 0.2–1.0 μg.kg^−1^h^−1^. The co-administration of midazolam and fentanyl in the DMF group did not decrease the MBP as compared with the baseline value, except for a significant decrease in MBP by 10.4 % only 3 min after the start of the drug administration. By contrast, the ketamine co-administered in the DK group significantly increased the MBP by 15 % as compared with the baseline value 3 and 6 min after the start of the drug administration, and did not show significant changes thereafter. In terms of HR change, both groups showed significant decreases by 12–20 % as compared with the baseline value throughout the study, except for a lack of change only 3 min after the start of the drug administration in the DK group. Those levels of hemodynamic changes were within the clinically acceptable range. In addition, the number of patients requiring ephedrine for hypotension and atropine for bradycardia was 10 of 52 (7 of 25 in the DK group and 3 of 27 in the DMF group) and 3 of 52 (in the DK group only), respectively, and the patients recovered easily after treatment. These differences were not statistically significant between the two groups. Taking the results in the hemodynamic changes mentioned above, we suggest that the administration of ketamine or midazolam-fentanyl as dexmedetomidine adjuvants is safe in terms of hemodynamic stability.

The great advantage of dexmedetomidine for procedural sedation or sedation in the intensive care unit is the lack of respiratory depression [2, 20, 21]. There was no evidence of respiratory depression in the present study despite the co-administration of midazolam-fentanyl in the DMF group. This could be attributed to the fact that the use of a small dose of midazolam and fentanyl was possible as dexmedetomidine was the mainstream drug used for the sedation, and a local infiltration of lidocaine was also performed in this study.

In a study of conscious sedation for colonoscopy, the dexmedetomidine group showed a prolonged recovery time (mean = 39 min) as compared with the midazolam-meperidine (20 min) or fentanyl (17 min) groups [2]. Koruk et al. reported that pediatric patients undergoing extracorporeal shock wave lithotripsy showed prolonged recovery time after dexmedetomidine-ketamine sedation compared midazolam-ketamine sedation [7]. In our study, both the DK and DMF groups showed a similar mean time to spontaneous eye opening (around 7 min) and mean recovery room stay length (around 11–12 min), which were shorter than in the above-mentioned study. The difference between the previous results [2, 7] may be attributed to the types of procedures, the subjects, and the drug combinations.

The anterograde amnesia and anxiolytic action of midazolam may contribute to better sedation quality and patient satisfaction scores in DMF group. However, using midazolam is not always ensure the improvement of patient satisfaction. Dere et al. reported that midazolam-fentanyl group showed lower satisfaction scores compared dexmedatomidine-fentanyl group for conscious sedation [22]. Thus, it is essential that the appropriate combination of drugs for individual procedure and careful adjustment of the dose is required to improve patient satisfaction.

This study had several limitations. First, we did not enrolled elderly or critically ill patients. Forty three out of fifty two patients were breast cancer patients, and forty one out of fifty two patients were ASA status I, and the average age of the patients was around 50 years. Second, the anesthesiologist were not blinded to the group assignment for titration of the drugs and management of the adverse effects. Third, the present study shows large standard deviations in recovery time, so it may have been underpowered to detect the differences in recovery time. Thus, further studies are needed to determine the best combination of analgo-sedative drugs and the appropriate dosage of drugs to avoid adverse effects in elderly or critically ill patients.

## Conclusions

In conclusion, both ketamine and midazolam-fentanyl co-administration with dexmedetomidine for MAC showed comparable recovery time, onset time, cardiorespiratory variables, and analgesia. However, the dexmedetomidine-midazolam-fentanyl combination showed a better sedation quality and satisfaction scores despite the lower infusion rate of dexmedetomidine, and a higher incidence of BIS < 60 than the dexmedetomidine-ketamine combination.

## Abbreviations

ASA, American Society of Anesthesiologists; BIS, bispectral index; BP, blood pressure; DK, dexmedetomidine-ketamine; DMF, dexmedetomidine-midazolam-fentanyl; HR, heart rate; IV, intravenous; MAC, monitored anesthesia care; MBP, mean blood pressure; OAA/S, the modified Observer’s Assessment of Alertness/Sedation; RR, respiratory rate; SD, standard deviation; SpO_2_, oxygen saturation by pulse oximeter
